# Transcriptional Profiling Reveals the Regulatory Role of DNER in Promoting Pancreatic Neuroendocrine Neoplasms

**DOI:** 10.3389/fgene.2020.587402

**Published:** 2020-11-27

**Authors:** Rui He, Wunai Zhang, Shuo Chen, Yang Liu, Wenbin Yang, Junhui Li

**Affiliations:** ^1^Department of Oncology, The Second Affiliated Hospital of Xi’an Jiaotong University, Xi’an, China; ^2^Department of General Surgery, The Second Affiliated Hospital of Xi’an Jiaotong University, Xi’an, China; ^3^Department of Hepatobiliary Surgery, The First Affiliated Hospital of Xi’an Jiaotong University, Xi’an, China

**Keywords:** pancreatic neuroendocrine neoplasms, RNA sequencing, DNER, Wnt, Notch

## Abstract

Wnt/β-catenin and NOTCH signaling contribute to the pathogenesis and growth of (PanNENs). The wnt and Notch signaling pathways form an integrated signaling device termed “wntch” and regulate stochastic cell fate decisions, suggesting the essentiality of Wnt/Notch interactions in disease progression. However, the function of Wnt/Notch interactions in PanNENs is unclear. We analyzed RNA sequencing (RNA-seq) data to identify differentially expressed lncRNAs, mRNAs and pathways according to enriched Gene Ontology (GO) terms and Kyoto Encyclopedia of Genes and Genomes (KEGG) pathways associated with PanNENs. RNA-seq analysis revealed that the levels of the lncRNA XLOC_221242 and the mRNA encoding Delta/Notch-like epidermal growth factor (EGF)-related receptor (DNER) were significantly increased in tumor tissues compared with normal tissues (*n* = 3). Protein-protein interaction (PPI) prediction combined with transcriptional profiling data analysis revealed that DNER expression levels were positively correlated with those of DNA-binding factor (RBPJ), S phase kinase-associated protein 1 (Skp1), CTNNB1 and Cadherin-2 (CDH2), which promote PanNEN tumorigenesis and progression. These results were consistent with those of immunohistochemical analysis of DNER, RBPJ, SKP1, CTNNB1, and CDH2 expression (*n* = 15). These findings provide compelling clinical and molecular evidence supporting the conclusion that DNER and the related RBPJ, SKP1, CTNNB1, and CDH2 signaling contribute to PanNEN tumorigenesis and progression by activating wnt/Notch interactions.

## Introduction

The incidence of pancreatic neuroendocrine neoplasms (PanNENs), which account for only 1–2% of all pancreatic malignancies, is increasing (Anderson and Bennett, 2016). The treatment, curative surgery and systemic medication, is dependent on various factors to improve the survival outcome of patients with PanNENs (Ishida and Lam, 2020). The survival of patients with PanNENs has improved over time, but some factors, including G stage, surgical resection margin, lymph node, TMN stage, metastasis, the necrosis and vascular invasion (Gao Y. et al., 2018). Although most PanNENs are benign, some can progress to metastasis, depending on their size, functional status, and grade (Fang and Shi, 2019).

The molecular pathogenesis of PanNENs is not clear. Via whole-exome sequencing, MEN1 inactivation, DAXX/ATRX mutation and/or loss, and mutations in mammalian target of rapamycin (mTOR) pathway genes, MUTYH, CHECK2, and BRCA2 were found to be related to the tumorigenesis of PanNENs (Vinik et al., 2000; Chai et al., 2018). To explore lncRNAs in PanNENs, sequencing is used. High expression of either of two lncRNAs—MALAT1 or HOTAIR—was reported to be negatively associated with characteristics of lower aggressiveness, including lower T stage and less frequent development of metastases, independent of histologic grade (Chu et al., 2019).

The functional roles of long noncoding RNA (lncRNA)-mRNA expression profiles in PanNENs are unclear. This study aimed to identify the lncRNA and mRNA expression profiles and explore the lncRNA-mRNA coexpression networks associated with PanNEN tumorigenesis.

## Materials and Methods

### Patients and Samples

Between September 2010 and December 2019, we obtained 15 samples of pathologically proven PanNEN tissues from patients at the Second Affiliated Hospital, Xi’an Jiaotong University. Then, three pairs of samples (including tumor and adjacent tumor tissues) from five random patients were used for RNA sequencing (RNA-seq) analysis. All samples were frozen in liquid nitrogen immediately after resection and stored at −80°C.

### RNA-Seq and Data Analysis

Total RNA was extracted using TRIzol reagent (Takara Biomedical Technology, Beijing, China). The RNA integrity and quantity were finally measured using an RNA Nano 6000 Assay Kit in a Bioanalyzer 2100 system (Agilent Technologies, CA, United States). The RNA library for lncRNA-seq was constructed as a stranded library with rRNA depletion. After library construction and sample pooling, the samples were subjected to Illumina sequencing. lncRNA-seq commonly uses PE150 (paired-end 150 nt) sequencing to obtain 12G of raw data. Raw data (raw reads) in FASTQ format were first processed with in-house Perl scripts. The clean reads for each sample were first mapped to a reference genome with HISAT2 software. The read alignment results were then transferred to the program StringTie for transcript assembly. All transcripts were merged using Cuffmerge software. The characteristics of novel lncRNAs were compared with those of known lncRNAs and mRNAs. Quantification of transcripts and genes was performed using StringTie software, and Reads Per Kilobase of transcript per Million mapped reads (RPKM) values were obtained. The resulting *P*-values were adjusted using the Benjamini and Hochberg method for controlling the false discovery rate (FDR). Genes meeting the following criteria were considered differentially expressed: | log2 (fold change) | >0 and adjusted *P*-value (padj) <0.05.

### GO Enrichment, KEGG Pathway, and PPI Analyses

The functional roles of differentially expressed genes (DEGs) were revealed by determining the transcriptional profiles acquired using RNA-seq and by Gene Ontology (GO) and Kyoto Encyclopedia of Genes and Genomes (KEGG) enrichment analyses. GO covers three domains: cellular component (CC), molecular function (MF), and biological process (BP). The KEGG resource is a collection of databases encompassing genomes, biological pathways, diseases, drugs, and chemical substances. The KEGG pathway database, the wiring diagram database, is the core of the KEGG resource. It is a collection of pathway maps integrating many entities, including genes, proteins, RNAs, chemical compounds, glycans, and chemical reactions, as well as disease genes and drug targets, which are stored as individual entries in the other KEGG databases. The PPI network of the DEGs was constructed according to information acquired using the STRING database^1^. To identify hub genes in the PPI network, we implemented maximal clique centrality analysis into cytoHubba (a Cytoscape plugin). Maximal clique centrality is a topological analytical method that effectively screens for hub genes (Chin et al., 2014).

### LncRNA-mRNA Coexpression Network Construction

To explore interactions among these differential expression profiles of lncRNAs and mRNAs in PNEN tumors and adjacent tissues, we constructed coexpression network. The lncRNA-mRNA networks were constructed according to the normalized signal intensity of specific mRNA and lncRNA expression. For each mRNA-lncRNA pair, we calculated the Pearson correlation coefficient and selected the significant correlation pairs used to construct the network. In network analysis, degree centrality is the key parameter that determines the relative importance of an mRNA or lncRNA within the network19. Degree centrality is defined as the number of links numbers between one node and another node. The clustering coefficient indicates the density of the connections between each gene and the adjacent genes; the larger the clustering coefficient, the greater is the importance of a gene in regulating the network.

### Immunohistochemistry (IHC)

Immunohistochemical staining was performed on 3-μm sections of formalin-fixed, paraffin-embedded samples. After deparaffinization with xylene and rehydration, antigen retrieval was performed with 10 mM citrate buffer (pH 6.0) for 20 min. Endogenous peroxidase activity was blocked by incubation with 0.3% hydrogen peroxide and a blocking protein (Dako, CA, United States) for 10 min. The primary antibody was added overnight at 4°C, and the sections were then washed. The following antibodies were used: mouse monoclonal anti-Delta/Notch-like epidermal growth factor (EGF)-related receptor (DNER) (Merck Life Science (Shanghai) Co., Ltd. Shanghai, China), rabbit anti-DNA-binding factor (RBPJ) (Merck Life Science (Shanghai) Co., Ltd., Shanghai, China), rabbit anti-S phase kinase-associated protein 1 (SKP1) (Merck Life Science (Shanghai) Co., Ltd., Shanghai, China), rabbit anti-CTNNB1 (Merck Life Science (Shanghai) Co., Ltd., Shanghai, China), and mouse monoclonal anti-Cadherin-2 (CDH2) (Merck Life Science (Shanghai) Co., Ltd., Shanghai, China). The secondary biotinylated goat anti-mouse or goat anti-rabbit antibody (Dako) was then applied for 30 min, and the sections were then incubated with streptavidin peroxidase (Dako LSAB+ HRP kit) for 30 min. After washing, the slides were stained with diaminobenzidine (DAB) chromogen solution (Dako), counterstained with hematoxylin in accordance with a standard protocol, dehydrated through a graded ethanol series, and mounted. Sections were without the primary antibodies to ensure the specificity of the primary antibodies. No immunostaining was detected in these sections, indicating the specificity of the primary antibodies used in this study.

### Statistical Analysis

The Raw data meet the standard for the next step by assessing sequencing error rate, data volume, comparison rate, etc. The RNA-seq standard analysis process includes quality control, comparison, splicing, screening, quantitative analysis, difference significance analysis, and functional enrichment.

Quality assessment of sequencing data, including original sequence data, sequencing data filtering, sequencing error rate distribution check, GC content distribution difference, and clean reads used for subsequent analysis were obtained. StringTie applied the network flow algorithm to splice and quantify transcripts and genes. Cuffmerge software was used to merge the transcripts obtained from the splicing of various samples. The comparison, splicing, and screening of the known transcripts and the predicted Novel_lncRNA, Novel_mRNA, and Unclassified transcripts were all quantified with expression levels. After the quantitative analysis is completed, the expression matrix of all samples can be obtained, and then the expression significance analysis of gene or transcript level can be performed in order to find the functional genes or transcripts related to the treatment group. The gene was analyzed with Cuffdiff (quantified with Cuffdiff) or edgeR (quantified with RSEM) software, and padj <0.05 was taken as the significance criterion for the difference (if PADJ was less than 0.05, *P*-value < 0.05 was used for the difference screening).

## Results

### DEGs Associated With PanNENs

**FIGURE 1:**
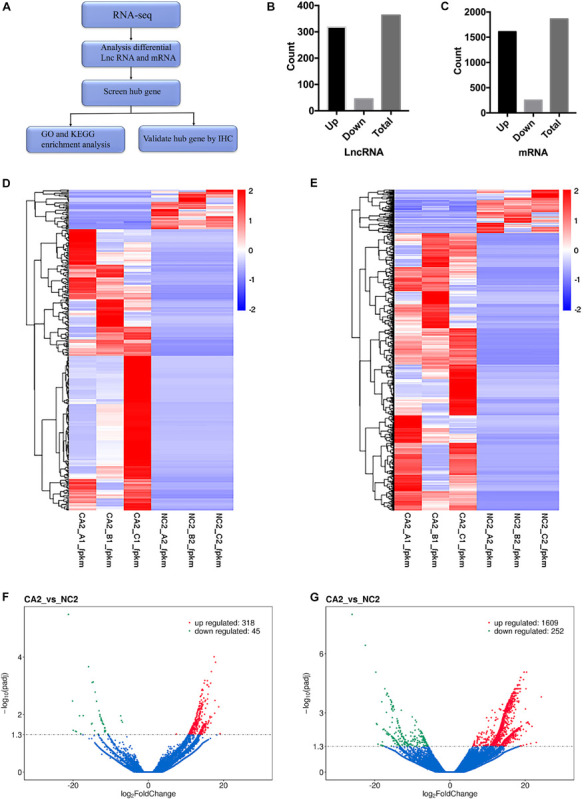
Differential lncRNA and mRNA expression between tumor and paratumor tissues of patients with PanNENs. **(A)** Workflow of the study. **(B)** Numbers of upregulated and downregulated lncRNAs. **(C)** Numbers of upregulated and downregulated mRNAs. **(D)** Heatmap of differentially expressed lncRNAs between the tumor and paratumor tissues. **(E)** Heatmap of differentially expressed mRNAs between the tumor and paratumor tissues. **(F)** Volcano plot of differentially expressed lncRNAs between the tumor and paratumor tissues. **(G)** Volcano plot of differentially expressed mRNAs between the tumor and paratumor tissues *n* = 3.

The workflow of the study is shown in Figure 1A. To identify DEGs, we performed RNA-seq on samples from three patients with PanNENs. We identified 363 differentially expressed lncRNAs, of which 318 were upregulated and 45 were downregulated at the transcriptional level (Figure 1B). We also identified 1861 differentially expressed mRNAs, of which 1609 were upregulated and 252 were downregulated (Figure 1C). The top 20 differentially expressed lncRNAs between the normal and tumor tissues of patients with PanNENs are listed in Table 1. The top 20 differentially expressed mRNAs (i.e., DEGs) between the normal and tumor tissues of patients with PanNENs are listed in Table 2. Heatmaps and volcano plots of the distributions of differentially expressed lncRNAs and mRNAs are shown in Figures 1D,E. Volcano plots of the distributions of DEGs are shown in Figures 1F,G. The lncRNA XLOC_221242 was one of the most upregulated lncRNAs among tumor tissues compared with the matched paratumor tissues (log2 fold change = 13.8463, padj = 0.002372158).

**TABLE 1:** Patient characteristics.

	**Overall (*n* = 16)**	**PNET (*n* = 11)**	**PNEC (*n* = 5)**
Age, median (range)	55.4(35−68)	54.3(35−65)	58.0(49−68)
Gender, no. (%)			
°Male	9 (56.3)	6 (54.5)	2 (40)
°Female	7 (43.7)	5 (45.5)	3 (60)
°Tumor size (cm)	3.375	3.45	3.2
WHO classification, no. (%)			
°NET G1	1 (6.25)	1 (9.1)	
°NET G2	10 (62.5)	10 (90.9)	
°NET G3	5 (31.25)		5 (100)
° (NEC) Unknown	0	0	0
Curative resection, no. (%)			
°Yes	12 (75)	8 (72.7)	4 (80)
°No	4 (25)	3 (27.3)	1 (20)
Disease sites, no. (%)			
°Head	4 (25)	2 (18.18)	2 (40)
°Neck	11 (68.75)	0 (0)	1 (20)
°Body and tail	1 (6.25)	9 (81.82)	2 (40)
Ki-67 index (%)			
°≤2%	3 (18.75)	3 (27.27)	0 (0)
°3-20%	11 (68.75)	8 (72.73)	3 (60)
°>20%	2 (12.5)	0 (0)	2 (40)
CgA (%)			
°Positive	16 (100)	11 (100)	5 (100)
°Negtive	0 (0)	0 (0)	0 (0)

**TABLE 2:** Top 20 differentially expressed LncRNA between tumor tissues and normal tissues of patients with PanNENs.

**Gene_id**	**Gene name**	**CA2_FPKM**	**NC2_FPKM**	**Log2Fold change**	***P*-adj**	**UP/Down**
XLOC_221242	XLOC_221242	43.868717	0	13.84633451	0.002372158	UP
XLOC_001021	XLOC_001021	30.916481	0	19.08556019	0.045747422	UP
XLOC_000438	XLOC_000438	26.02661	0	18.73528684	0.005385522	UP
XLOC_007895	XLOC_007895	13.885583	0	15.94233185	0.019183975	UP
XLOC_121259	XLOC_121259	5.570594333	0	14.12548719	0.036679241	UP
XLOC_121456	XLOC_121456	4.664696	0	15.22911587	0.006009066	UP
ENSG00000283217	AC068205.1	4.406602667	0	13.12333025	0.021655539	UP
XLOC_185143	XLOC_185143	4.323860333	0	15.75083787	0.028367132	UP
XLOC_000502	XLOC_000502	2.878419667	0	16.63851527	0.019756611	UP
ENSG00000243701	DUBR	2.863058	0	12.96832391	0.010042746	UP
ENSG00000130669	PAK4	6.864150667	534.4499207	−7.343546907	0.011034912	Down
ENSG00000089163	SIRT4	3.414264667	194.723648	−6.902059562	0.018306064	Down
ENSG00000132906	CASP9	1.585171333	106.159612	−7.140516249	0.016036468	Down
ENSG00000174876	AMY1B	0	56.44988633	−21.25643039	3.26E-06	Down
XLOC_001021	XLOC_001021	0	36.775833	−19.35332279	0.037935633	Down
XLOC_000423	XLOC_000423	0	35.82703133	−20.15640705	0.003373516	Down
XLOC_000438	XLOC_000438	0	32.291636	−19.98628759	0.035141067	Down
XLOC_001021	XLOC_001021	0	16.51662433	−19.12719458	0.039133681	Down
XLOC_000423	XLOC_000423	0	9.269675333	−18.30626306	0.01095923	Down
XLOC_001021	XLOC_001021	0	7.757990667	−17.96437169	0.045899957	Down

### GO and KEGG Analyses of DEGs

To further illustrate the functions of DEGs in PanNENs, GO and KEGG pathway enrichment analyses were performed. The clusterProfiler^2^ software was used to explore the pathway enrichment analysis with gene database of GO and KEGG. From the GO enrichment analysis results, the most significant 20 terms were selected to draw the histogram for display and Padj < 0.05 was the significant enrichment of KEGG pathway. GO analysis identified 267 GO terms significantly enriched with the DEGs: 44 MF terms, 167 BP terms and 56 CC terms. Synaptic transmission, neuron projection, synapse and cell projection part were the four descriptive terms (Supplementary Figure 2A). The top 20 significantly enriched GO terms in the BP, CC and MF categories are shown in Supplementary Figures 2B–D, respectively. BP analysis showed that the top two terms enriched with DEGs were related to synaptic transmission (*n* = 216) and potassium ion transport (*n* = 65). CC analysis showed that the top two terms enriched with DEGs were neuron projection (*n* = 222) and synapse (*n* = 192). MF analysis showed that the top two terms enriched with DEGs were gated channel activity (*n* = 109) and ion channel activity (*n* = 119). Among these terms, neuron projection (*n* = 222) and synaptic transmission (*n* = 216) were the most highly enriched with DEGs. Three pathways with padj <0.05 were identified by KEGG pathway analysis of the DEGs, as shown in Supplementary Figures 2E,F. Among these pathways, the most significantly enriched were neuroactive ligand-receptor interaction (*n* = 83) and olfactory transduction (*n* = 78). It is not clear weather GO and KEGG pathway enrichment analyses is related to the expression level of genes among tumor tissues compared with the matched paratumor tissues.

### Construction of lncRNA-mRNA Coexpression Networks and PPI Network in PanNENs

To analyze protein interactions, a protein-protein interaction (PPI) network was constructed with the STRING online database. We have selected the 100 upregulated mRNAs identified in the above RNA-seq analysis to construct the PPI network. Network analysis revealed that the DNER network was mainly involved in regulating the Wnt and Notch signaling pathways required for cell proliferation, differentiation and apoptosis (Supplementary Figure 3A). PPI analysis indicated that the levels of the mRNAs encoding RBPJ, SKP1, CTNNB1, CDH2, SCG2, VGF, and TFF3 were significantly increased in PanNEN tumor tissues compared with the matched paratumor tissues and may be involved in the DNER signaling pathway in PanNENs (Supplementary Figure 3A). Comparing PPI results with GO and KEGG results, we found that DNER signaling pathway participating in many processes of GO and KEGG pathway enrichment, including secretion by cell, cytoplasmic vesicle and calcium ion binding.

The coexpression networks in the tumor and paratumor tissue were constructed according to the normalized signal intensity of specific mRNA and lncRNA expression. Due to the abundant information in the mRNA-lncRNA coexpression network, we sorted the mRNAs/lncRNAs by their degree, which represents the number of interactions with other mRNAs/lncRNAs in the network, selected the top five lncRNAs and top 100 mRNAs, and constructed the coexpression network related to these genes. In Supplementary Figure 3B, the red circles represent lncRNAs, the blue ovals represent mRNAs, and the lines represent functional relationships. The 100 upregulated mRNAs were matched with thefive5 upregulated lncRNAs in the coexpression network table. We found that three of the five lncRNAs were coexpressed with 12 of the 100 mRNAs and that DNER was the most upregulated mRNA.

#### Patient Characteristics

A total of 16 patients with PanNENs were enrolled in this study (Table 1). The median age was 55.4 (range, 35–68), and the cohort had a male:female ratio of 9:7. The vast majority of the patients (74%) had nonfunctioning tumors. Eleven patients were diagnosed with PanNENs, and five patients were diagnosed with pancreatic neuroendocrine carcinoma (PNEC). Among the 16 patients, the mean tumor diameters were 3.375, 3.45, and 3.20 cm in the combined cohort, patients with PanNEN and patients with PNEC, respectively. According to the WHO classification criteria, 1/11 patients had G1, 10/11 had G2 and 5 had G3 tumors. Among the patients, 8/11 (72.7%) in the PNEN group and 4/5 (80%) in the PNEC group underwent curative resection. Among the 11 PNEN patients, 2/11 (18.18%) had a tumor localized at the head of the pancreas, and 9/11 (81.82%) had a tumor localized at the body or tail of the pancreas. Among the five PNEC patients, 2/5 (40%) had a tumor localized at the head of the pancreas, 1/5 (20%) had a tumor localized at the neck of the pancreas and 2/5 (40%) had a tumor localized at the body or tail of the pancreas. The Ki-67 index was also analyzed. Among the 11 PNEN patients, the Ki-67 index was ≤2% in 3/11 (27.27%) and 3–20% in 8/11 (72.73%). Among the five PNEC patients, the Ki-67 index was 3–20% in 3/5 (60%) and >20% in 2/5 (40%). We next analyzed the CgA status and found that all patients were positive.

### High Expression Levels of DNER and Related Genes in PanNETs

**FIGURE 2:**
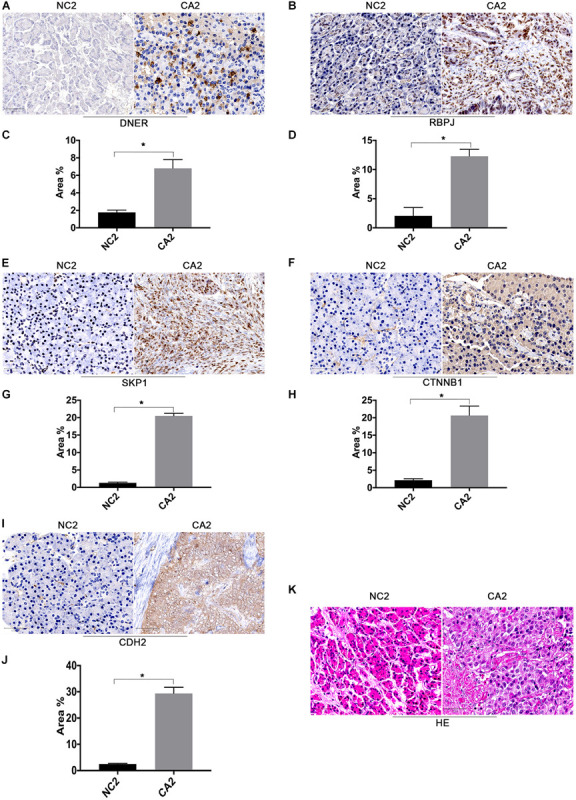
Expression levels of DNER signaling pathway components in the clinical cohort. **(A)** Immunohistochemical staining of DNER in PanNEN tumor tissues compared with matched paratumor tissues. **(B)** Immunohistochemical staining of RBPJ in PanNEN tumor tissues compared with matched paratumor tissues. **(C)** Immunohistochemical staining analysis with IMAGE J was used to determine the protein level of DNER. **(D)** Immunohistochemical staining analysis with IMAGE J was used to determine the protein level of RBPJ. **(E)** Immunohistochemical staining of SKP1 in PanNEN tumor tissues compared with matched paratumor tissues. **(F)** Immunohistochemical staining of CTNNB1 in PanNEN tumor tissues compared with matched paratumor tissues. **(G)** Immunohistochemical staining analysis with IMAGE J was used to determine the protein level of SKP1. **(H)** Immunohistochemical staining analysis with IMAGE J was used to determine the protein level of CTNNB1. **(I)** Immunohistochemical staining analysis with IMAGE J was used to determine the protein level of CDH2. **(J)** Immunohistochemical staining analysis with IMAGE J was used to determine the protein level of CDH2. **(K)** Hematoxylin-eosin (HE) staining in PanNEN tumor tissues compared with matched paratumor tissues. (*P < 0.05 compared with paratumor tissues).

We validated the DNER expression levels in cohort two. The RNA-seq profiles revealed that the levels of DNER mRNA and coexpression of LncRNA XLOC_221242 in tumors, compared with those of normal tissues, were significantly increased in pancreatic neuroendocrine tumor (PNET) patients. PPI analysis indicated that the levels of the mRNAs encoding RBPJ, SKP1, CTNNB1, and CDH2 were significantly increased and that these molecules may be involved in the DNER signaling pathway. We then quantified the expression of DNER, RBPJ, SKP1, CTNNB1, and CDH2 in solid tumor and paratumor paraffin sections by immunohistochemistry and found strong cytoplasmic positivity of DNER in tumor tissue compared with paratumor tissue (Figure 2A), with significant differences (Figure 2C, *P* < 0.05). We also found strong nuclear positivity of RBPJ and SKP1 in tumor tissue compared with paratumor tissue (Figures 2B,E), with significant differences (Figures 2D,G, *P* < 0.05). CTNNB1 and CDH2 were localized at the cell membrane, and we found strong cell membrane positivity of CTNNB1 and CDH2 in tumor tissue compared with paratumor tissue (Figures 2F,I), with significant differences (Figures 2H,J, *P* < 0.05). The structure of the tumor and paratumor tissue is shown in Figure 2K.

**FIGURE 3:**
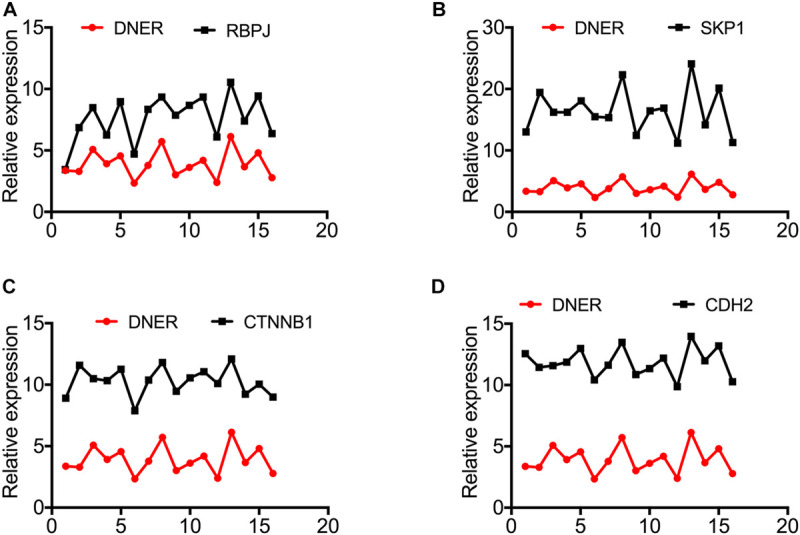
The expression levels of RBPJ, SKP1, CTNNB1, and CDH2 are upregulated and positively correlate with DNER expression in PanNENs. **(A)** RBPJ protein expression compared with DNER protein expression in PanNENs (*n* = 15). **(B)** SKP1 protein expression compared with DNER protein expression in PanNENs (*n* = 15). **(C)** CTNNB1 protein expression compared with DNER protein expression in PanNENs (*n* = 15). **(D)** CDH2 protein expression compared with DNER protein expression in PanNENs (*n* = 15).

**FIGURE 4:**
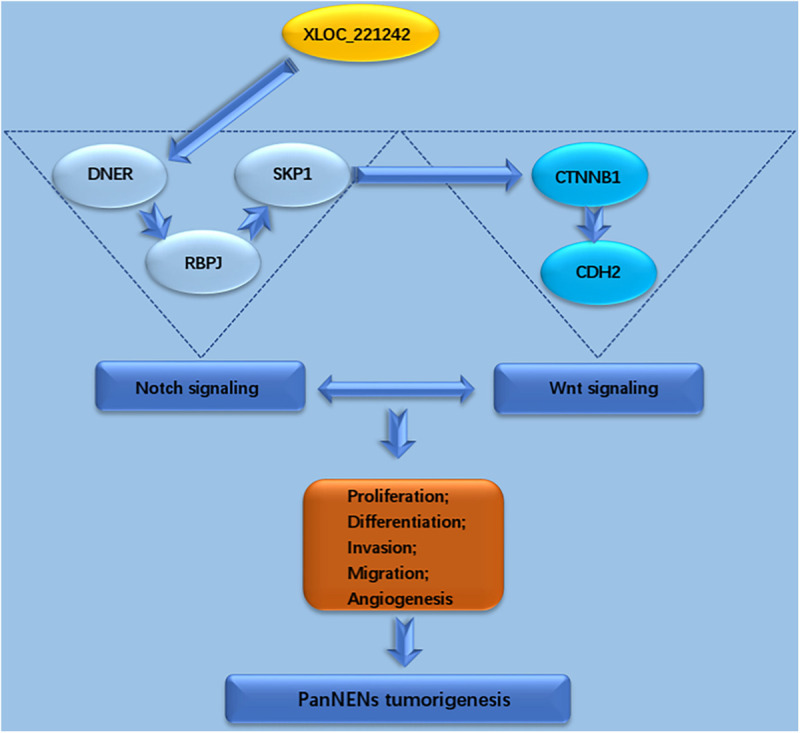
LncRNA XLOC_221242 regulates the expression of DNER, which is the key factor of Notch signal pathway. DNER regulates its downstream target RBPJ and SKP1, and also interacts with the important factors of Wnt signaling pathways, CTNNB1 and CDH2, regulating tumor proliferation, differentiation, invasion, migration, and angiogenesis of PanNENs tumorigenesis.

Moreover, the expression levels of RBPJ, SKP1, CTNNB1, and CDH2 were positively correlated with those of DNER (Figures 3A–D). Taken together, these findings indicate that DNER may upregulate the expression of RBPJ, SKP1, CTNNB1, and CDH2 to promote PNEN tumorigenesis. We made a model of the pathway of PanNENs. LncRNA XLOC_221242 regulates the expression of DNER, which is the key factor of Notch signal pathway. DNER regulates its downstream target RBPJ and SKP1, and also interacts with the important factors of Wnt signaling pathways, CTNNB1 and CDH2, regulating tumor proliferation, differentiation, invasion, migration and angiogenesis of PanNENs tumorigenesis (Figure 4).

## Discussion

Due to the widespread use of imaging studies, the diagnosis of PanNENs has significantly increased. Most PanNENs can be diagnosed in asymptomatic, early or metastatic stages. PanNENs can be classified according to their Ki-67 index and can be classified as functional or nonfunctional depending on the clinical or hormonal hypersecretion syndrome (Larghi et al., 2019). Surgical treatment is the main therapeutic method and can significantly prolong life expectancy (Szeliga and Jackowski, 2018). The survival of patients with all NETs, especially those with distant-stage gastrointestinal NETs and PanNENs, has improved over time, reflecting improvements in therapies, which will help us to prioritize future research directions (Dasari et al., 2017).

A larger proportion of germline mutations are found in sporadic pancreatic neuroendocrine tumors (PanNETs) than in other NETs in genes such as the DNA repair genes *MUTYH*, *CHEK2*, and *BRCA2*, affecting processes such as chromatin remodeling, activation of mTOR signaling (including previously undescribed *EWSR1* gene fusions), telomere maintenance, hypoxia and HIF signaling (Scarpa et al., 2017). Proteomic analysis has also identified several regulator proteins with critical tumor dependencies, including key regulators of the neuroendocrine lineage progenitor state and immunoevasion (Alvarez et al., 2018). Moreover, numerous studies have investigated the prognostic utility of epigenetic profiles and tissue-based biomarkers as well as the expression of various noncoding RNAs, including lncRNAs and microRNAs (miRNAs) (Lee et al., 2019).

In this study, high-throughput RNA-seq technology designed for genome-wide identification was used to detect the profiles of differentially expressed lncRNAs and mRNAs in three pairs of tumors and corresponding para-tumor tissues in PanNENs. To screen clinically significant lncRNAs and mRNAs from the large number of differential transcripts, we screened the transcripts according to the following criteria: the same differential expression in all three pairs of samples and higher absolute fold change values o as selected by coexpression network analysis. Finally, we used IHC to select 5 coexpressed proteins in 15 PanNEN samples.

Aberrant mutation or epigenetic silencing of Wnt/β-catenin contributes to the pathogenesis and growth of NETs and has important clinical implications for their prognosis and treatment (Kim et al., 2013). The Wnt/β-catenin pathway exhibits alterations, including β-catenin accumulation and loss of APC, in some PanNENs, and these alterations are not correlated with tumor grade, tumor stage, or disease-specific survival (Weiss et al., 2016). Neurotensin (NT) is a direct target of the Wnt/β-catenin pathway, and inhibition of NT signaling can suppress cell proliferation and decrease the expression levels of growth-related proteins in NET cells (Kim et al., 2015).

The NOTCH signaling pathway, which can act as either a tumor suppressor or an oncogene, has been shown to play an important role in the pathogenesis of NENs. NOTCH receptors can be found on neuroendocrine cells, suggesting the need to fully understand the role and potential therapeutic implications of gene mutations and NOTCH signaling in NENs (Von Arx et al., 2019). Moreover, the genetic heterogeneity of NETs suggests that a full understanding of the roles of Notch signaling in NETs is required (Crabtree et al., 2016).

The Notch and Wnt pathways are two highly conserved signaling pathways during animal development. The molecular mechanisms underpinning the interactions between the Notch and Wnt pathways can cooperatively regulate transcriptional targets; in addition, one pathway can affect the other, and direct molecular crosstalk occurs between the signal transduction machineries (Collu et al., 2014). The wnt and Notch signaling pathways form an integrated signaling device termed “wntch” to limit variability in terms of sharpening boundaries and regulating stochastic cell fate decisions (Hayward et al., 2008). In both breast and colorectal cancer, wnt signaling can activate Notch by inducing Notch ligand expression, suggesting the essentiality of wnt/Notch interactions in disease progression (Rodilla et al., 2009; Lopez-Garcia et al., 2010).

GO analysis revealed the cell components, **BP**es and **MF**s in which the upregulated and downregulated mRNAs are involved in PanNEN. KEGG pathway analysis was applied to evaluate the differentially expressed mRNAs and cellular pathways. Collectively, the lncRNA-mRNA coexpression network and the – PPI network indicated that the levels of the mRNAs encoding RBPJ, SKP1, CTNNB1, CDH2, SCG2, VGF, and TFF3, which are related to NOTCH and Wnt signaling, were significantly increased in PanNEN tumor tissues compared with the matched paracancer tissues and that these molecules may be involved in the DNER signaling pathway in PanNENs.

Delta/Notch-like epidermal growth factor (EGF)-related receptor mediates Notch signaling via cell-cell interactions and contributes to the morphological and functional maturation of Bergmann glia via the Notch signaling pathway (Saito and Takeshima, 2006). DNER can also promote breast cancer cell progression and metastasis by activating Girdin/PI3K/AKT signaling (Wang et al., 2019). DNER promotes the PC-3 cell growth and prostate cancer progression by modulating the primary genes related to cancer stem cells (Wang et al., 2017). NOTCH1 regulates gene expression by associating with RBPJ and is oncogenic in murine and human T-cell progenitors (Wang et al., 2011). RBPJ is the major transcriptional effector of Notch signaling (Lake et al., 2014). Protein-bound polysaccharide-K (PSK) was found to reduce RBPJ expression and block RBPJ-induced invasiveness and proliferation under hypoxic conditions by inhibiting matrix metalloproteinase expression in pancreatic cancer cells (Yamasaki et al., 2016). Short hairpin RNA (shRNA) targeting RBPJ was found to efficiently inhibit cell growth and alter the expression of the cell cycle inhibitors p21 and p27; the cell cycle activators CDK2, CDK4, and CyclinD1; and the apoptosis suppressor Bcl-2 (Xue et al., 2015).

Skp1–Cullin1–F-Box protein (SCF) complexes play crucial roles in cellular processes and physiological dysfunctions such as those observed in cancer biology, including sustained proliferation, poor differentiation, invasion and migration, angiogenesis, DNA damage, metabolism, and resistance to cell death. Skp1 and SCF assembly components are coexpressed during the development of breast, colon, prostate, lung and gastric cancers (Hussain et al., 2016). The Skp1-6-OAP interaction results in proteolysis of NIPA, Skp2, and TRCP and upregulation of their substrates, such as E-Cadherin, ultimately resulting in prometaphase arrest (Liu et al., 2015). Cytoplasmic expression of SKP1 was found to be significantly associated with stratifin (SFN) positivity, tumor malignancy, and unfavorable patient outcomes. SFN-SKP1 binding results in SCF^*FBW7*^ dysfunction and allows several oncoproteins, including p-cyclin E1, p-c-Myc, p-c-Jun, and cleaved Notch 1, to evade ubiquitination and subsequent degradation (Shiba-Ishii et al., 2019).

CTNNB1 is a well-known member of the Wnt signaling pathway and encodes β-catenin. Polymorphisms in this gene are associated with cancer risk and may be novel predictive biomarkers for cancer risk (Li et al., 2017). β-Catenin is the main downstream effector and transcriptional coactivator of TCF/LEF target gene expression and is involved in canonical WNT signaling. WNT/CTNNB1 play a central role in the maintenance of multiple adult tissue stem cell populations, and their expression is a hallmark of colorectal cancer in early and later stages (Van Schie and Van Amerongen, 2020).

Cadherin-2 is a member of the cadherin family and regulates many cellular processes, including angiogenesis, apoptosis, and chemoresistance (Miao et al., 2018). CDH2 is upregulated in various cancers, including colorectal, bladder, lung and gastric cancers (Gao S. et al., 2018). miR-124 can inhibit the expression of CDH2, which might lead to a therapeutic strategy targeting CDH2 in human lung cancer (Ma et al., 2016). Activation of Wnt/β-catenin signaling can promote the expression of cadherin 2 and alter cell shape (Fu et al., 2020). After its production, SCG2 can be rapidly cleaved into bioactive peptides, including secretoneurin, which has been shown to suppress endothelial cell apoptosis and enhance the proliferation, migration and angiogenesis of endothelial cells by stimulating VEGF signaling, the MAPK system and/or the PI3-kinase/Akt pathway (Luo et al., 2020). SCG2 is upregulated in granulosa cells across species and may mediate ovulatory angiogenesis in the human ovary (Hannon et al., 2018).

RNA sequencing revealed the levels of DNER mRNA and coexpression of the lncRNA XLOC_221242 in tumors compared with normal tissues. PPI analysis indicated that the levels of the mRNAs encoding RBPJ, SKP1, CTNNB1, and CDH2 were significantly increased and that these molecules may be involved in the DNER signaling pathway. In addition, the expression levels of RBPJ, SKP1, CTNNB1, and CDH2 were positively correlated with those of DNER, as demonstrated by IHC. Taken together, these findings indicate that DNER may upregulate the expression of RBPJ, SKP1, CTNNB1, and CDH2 to promote PNEN tumorigenesis.

## Conclusion

Our study demonstrates that DNER may upregulate the expression of RBPJ, SKP1, CTNNB1, and CDH2 to promote PNEN tumorigenesis. Although our results are largely incomplete, these findings indicating the NOTCH-Wnt interaction may provide insight for PanNEN research, leading to an era of precision medicine for NETs.

## Data Availability Statement

The authors acknowledge that the data presented in this study must be deposited and made publicly available in an acceptable repository, prior to publication. Frontiers cannot accept a manuscript that does not adhere to our open data policies.

## Ethics Statement

The studies involving human participants were reviewed and approved by the Research Ethics Committee of The Second Affiliated Hospital, Xi’an Jiaotong University. The patients/participants provided their written informed consent to participate in this study. Written informed consent was obtained from the individual(s) for the publication of any potentially identifiable images or data included in this article.

## Author Contributions

RH and WZ made substantial contributions to the conception and design of the study. WZ, SC, YL, WY, and JL were involved in data acquisition, analysis, and interpretation. JL, WY, and RH drafted the article and critically revised it for important intellectual content. RH and WZ performed immunohistochemistry staining and analyzed the related data. All authors have read and approved the final manuscript.

## Conflict of Interest

The authors declare that the research was conducted in the absence of any commercial or financial relationships that could be construed as a potential conflict of interest.

## Footnotes

1 https://string-db.org/

2 http://www.bioconductor.org/packages/release/bioc/html/clusterProfiler.html

## Supplementary Material

Supplementary Figure 1 Gene ontology enrichment analysis and KEGG pathway analysis of upregulated mRNAs. The results of functional enrichment analysis in PanNEN tumor tissues compared with paratumor tissues are shown. **(A)** Top 20 significant terms. The red dots indicate the −log10 FDR value for each term. **(B)** GO BP analysis of upregulated mRNAs. **(C)** GO CC analysis of upregulated mRNAs. **(D)** GO MF analysis of upregulated mRNAs. (BP, biological process; CC, cellular component; MF, molecular function). **(E)** Top 20 pathways associated with upregulated genes in PanNEN tumor tissues compared with paratumor tissues. **(F)** The three pathways with a padj of <0.05 as determined by KEGG pathway analysis in PanNEN tumor tissues compared with paratumor tissues.

Supplementary Figure 2 PPI network and lncRNA-mRNA coexpression network in PanNENs. **(A)** PPI analysis of 100 upregulated mRNAs indicated that the mRNAs encoding RBPJ, SKP1, CTNNB1, CDH2, SCG2, VGF, and TFF3 were significantly increased in PanNEN tumor tissues compared with the matched paratumor tissues and may be involved in the DNER signaling pathway in PanNENs. **(B)** Genes coexpressed with the top five lncRNAs and top 100 mRNAs in PanNEN tumor tissues were analyzed, and the coexpression networks are displayed. The red circles represent lncRNAs, the blue circles represent mRNAs and the lines represent functional relationships. The size of the circles represents the interaction degree of that gene product with other gene products.
