# Supplementary figures and images for: Transcriptional Profiling Reveals the Regulatory Role of DNER in Promoting Pancreatic Neuroendocrine Neoplasms
Source: Front Genet. 2020 Nov 27;11:587402. doi: 10.3389/fgene.2020.587402 (PMC7728999; doi:10.3389/fgene.2020.587402)

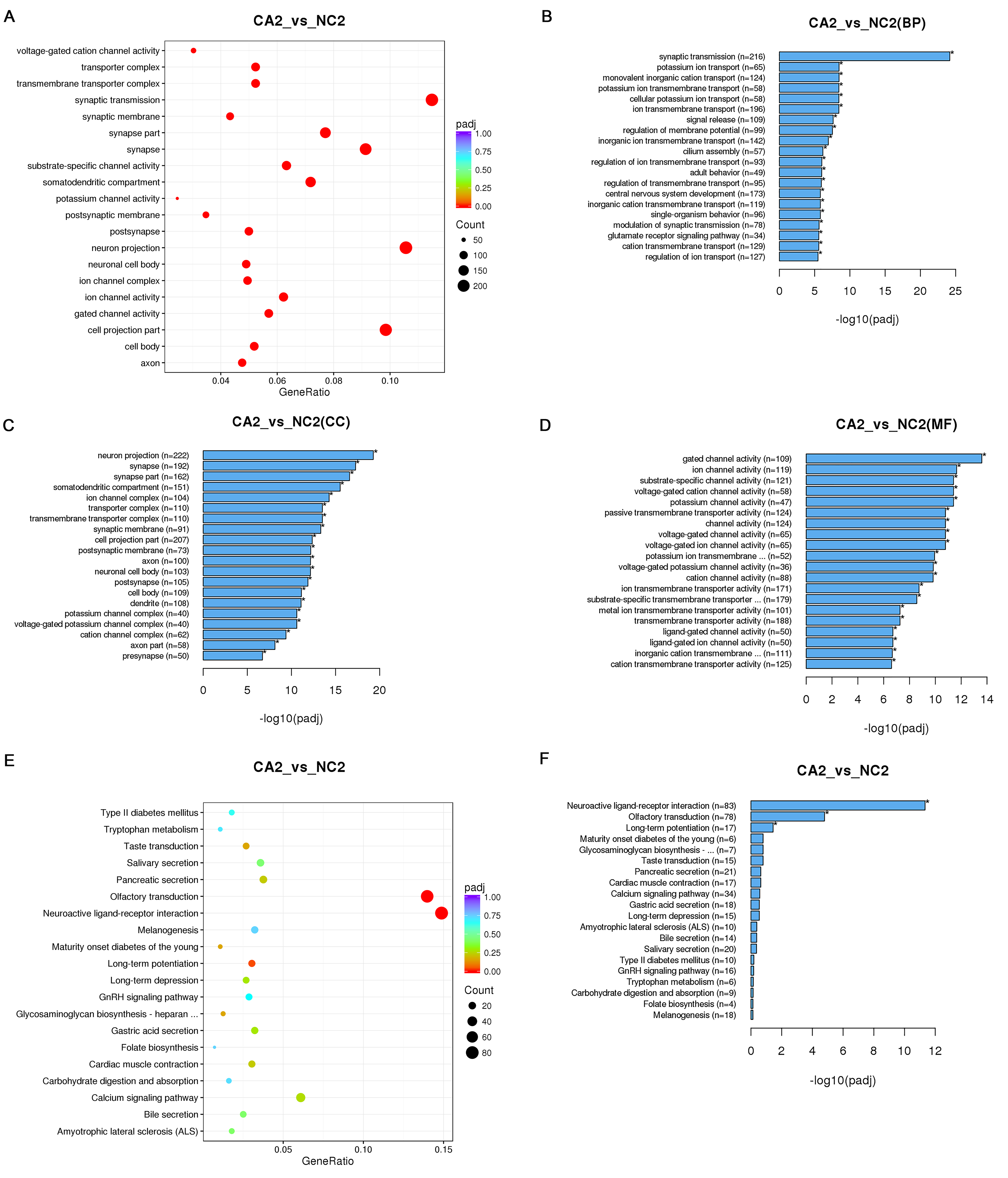

Supplement: Supplementary Figure 1 — Gene ontology enrichment analysis and KEGG pathway analysis of upregulated mRNAs. The results of functional enrichment analysis in PanNEN tumor tissues compared with paratumor tissues are shown. (A) Top 20 significant terms. The red dots indicate the −log10 FDR value for each term. (B) GO BP analysis of upregulated mRNAs. (C) GO CC analysis of upregulated mRNAs. (D) GO MF analysis of upregulated mRNAs. (BP, biological process; CC, cellular component; MF, molecular function). (E) Top 20 pathways associated with upregulated genes in PanNEN tumor tissues compared with paratumor tissues. (F) The three pathways with a padj of <0.05 as determined by KEGG pathway analysis in PanNEN tumor tissues compared with paratumor tissues. [file Image_1.TIF]

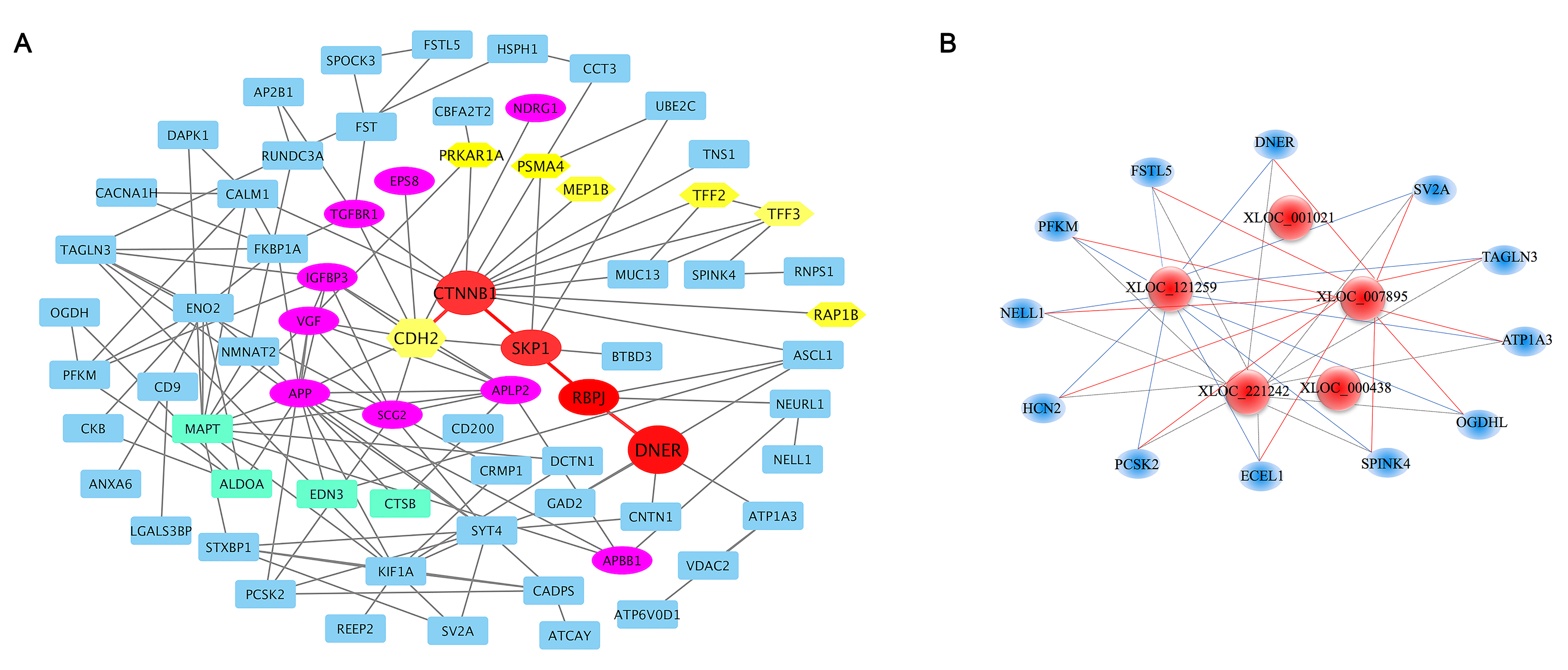

Supplement: Supplementary Figure 2 — PPI network and lncRNA-mRNA coexpression network in PanNENs. (A) PPI analysis of 100 upregulated mRNAs indicated that the mRNAs encoding RBPJ, SKP1, CTNNB1, CDH2, SCG2, VGF, and TFF3 were significantly increased in PanNEN tumor tissues compared with the matched paratumor tissues and may be involved in the DNER signaling pathway in PanNENs. (B) Genes coexpressed with the top five lncRNAs and top 100 mRNAs in PanNEN tumor tissues were analyzed, and the coexpression networks are displayed. The red circles represent lncRNAs, the blue circles represent mRNAs and the lines represent functional relationships. The size of the circles represents the interaction degree of that gene product with other gene products. [file Image_2.TIF]
